# TR35 Exerts Anti-tumor Effects by Modulating Mitogen-Activated Protein Kinase and STAT3 Signaling in Lung Cancer Cells

**DOI:** 10.3389/fcell.2021.723346

**Published:** 2021-10-25

**Authors:** Zhiyong Shi, Yang Gao, Lifeng Feng, Wencong Tian, Zhihua Dou, Chen Liu, Jie Liu, Yang Xu, Yachen Wang, Jie Yan, Qiang Wu, Jing Li, Liang Yang, Zhaocai Zhang, Jie Yang, Zhi Qi

**Affiliations:** ^1^Department of Molecular Pharmacology, School of Medicine, Nankai University, Tianjin, China; ^2^Department of Bioengineering, College of Life Science and Technology, Xinjiang University, Ürümqi, China; ^3^Key Laboratory of Emergency and Trauma of Ministry of Education, Research Unit of Island Emergency Medicine, School of Tropical Medicine and Laboratory Medicine, Chinese Academy of Medical Sciences (No. 2019RU013), Hainan Medical University, Haikou, China; ^4^Department of Critical Care Medicine, Second Affiliated Hospital, Zhejiang University School of Medicine, Hangzhou, China

**Keywords:** anti-tumor, camel milk, lung cancer, microenvironment, MAPK, STAT3

## Abstract

Cancer is a complex disease extremely dependent on its microenvironment and is highly regulated by a variety of stimuli inside and outside the cell. Evidence suggests that active camel whey fraction (TR35) confer anti-tumor effects in non-small cell lung cancer (NSCLC). However, its exact mechanisms remain elusive. Here, we investigated the mechanisms underlying suppression of NSCLC cell growth and proliferation by TR35. Treatment of A549 and H1299 cells with TR35 suppressed their growth and enhanced apoptosis, as revealed by CCK-8, colony formation and flow cytometric analyses. We find that TR35 suppresses tumor growth in a xenograft nude mouse model without losses in body weight. RNA-seq and KEGG pathway analyses showed that the DEGs were enriched in mitogen-activated protein kinase (MAPK) and Jak-STAT signaling pathways. After test the key factors’ activity associated with these pathways by Immunohistochemical (IHC) staining and western blotting, the activation of JNK phosphorylation and inhibition of p38 and STAT3 phosphorylation was observed both in TR35 treated lung cancer cell and tumor tissue. Taken together, these results showed that TR35 play a significant role in the NSCLC progression in the tumor microenvironment via MAPK and Jak-STAT signaling, highlighting TR35 as a potential therapeutic agent against lung cancer.

## Introduction

Lung cancer is a most common malignancy that is associated with high morbidity and mortality worldwide. Non-small cell lung cancer (NSCLC) accounts for 80–85% of lung cancers (No authors listed, 2020). Although treatment advances have significantly improved lung cancer prognosis, its 5-year survival remains low, at <15% (Miller et al., 2016), underscoring the need for effective preventive and therapeutic strategies against NSCLC.

In recent years, camel milk has been shown to be one of the most important special dairy products. Some dietary ingredients exhibit effectiveness against diabetes (Agrawal et al., 2005), hepatitis (El Miniawy et al., 2014), allergies (Shabo et al., 2005), autism, lactose intolerance (Shabo and Yagil, 2005), as well as cancer (Badawy et al., 2018; Kamal et al., 2018). In the traditional medicine of ethnic minorities in Xinjiang, camel milk has long been applied as an adjuvant anti-cancer treatment, including in lung cancer. Our previous study showed that, TR35, an active component of camel milk have inhibitory effects against esophageal cancer (Yang J. et al., 2019), but its effects against lung cancer are unclear.

The mitogen-activated protein kinase (MAPK) signaling cascade is made up of P38, JNK and ERK, and influences various processes, including cell proliferation, differentiation, apoptosis, and autophagy (Li et al., 2020). Numerous small molecule drugs and natural products inhibit cancer cell proliferation through MAPK signaling activation (Sui et al., 2017), highlighting its potential as an anti-cancer therapeutic target.

Signal transducer and activator of transcription 3 (STAT3) is overexpressed in various cancer types and modulates various cellular processes like proliferation, apoptosis, and differentiation, which makes it an attractive anticancer target (Subramaniam et al., 2013). Persistent STAT3 inhibition suppresses expression of its downstream targets, such as c-Myc, Bcl-XL, blocking cell cycle progression, and promoting apoptosis (Yu et al., 2007; Laudisi et al., 2018). STAT3 signaling is vital in lung cancer treatment (Bousquet Mur et al., 2020).

Here, we find that TR35 inhibits the growth and proliferation of lung cancer cells *in vitro* and *in vivo*. RNA-seq and Kyoto Encyclopedia of Genes and Genomes (KEGG) pathway analyses implied that TR35 may mediate its anti-cancer effects via MAPK and Jak-STAT signaling. Our data indicate that p-JNK, p-p38, and p-STAT3 might be key TR35 targets. Together, our findings highlight TR35 as a potential anti-NSCLC therapeutic factor.

## Materials and Methods

### Materials

TR35 was purified from Xinjiang Bactrian camel milk as previously described (Yang J. et al., 2019), dissolved in RPMI 1640 complete medium, and then filtered to remove bacteria.

### Cells Culture

H1299 and A549 (human lung cancer cell lines) were obtained from the Cell Bank of Shanghai. They were cultured in RPMI 1640 medium supplemented with 10% heat-inactivated FBS (BI, China) and 1% penicillin/streptomycin (Gibco, 10378016), in an incubator at 37°C, 5% CO_2_.

### Cell Cytotoxic Assay

Cell seeding was in a 96-well microplate at a density of 2,000 cells/well. Then, to allow attachment, they were cultured overnight. They were then treated with TR35 at 0, 1, 2, and 4 mg/ml for 24 and 48 h. To evaluate proliferation, 10 μL of CCK-8 (Dojindo, CK04-500) was added and cells incubated for 4 h. Next, absorbance was read at 450 nm after which we calculated the number of living cells per well. Each experiment was repeated at least three times.

### Colony Formation Assay

Cell seeding at a density of 500 cells/well was done in six-well plates, in triplicate. Then, they were cultured until visible colonies formed, with media change every 3 days. After 12 days, colonies were fixed in anhydrous methanol for a duration of 15 min, stained using 0.2% crystal violet (Sigma-Aldrich, C0775-25G), and the number of cells determined using a light microscope. The clone formation rate was given by the formula: $\mathrm{clone}\,\mathrm{formation}\,\mathrm{rate}\%=\frac{\mathrm{number}\,\mathrm{of}\,\mathrm{clones}}{\mathrm{number}\,\mathrm{of}\,\mathrm{inoculated}\,\mathrm{cells}}\times 100$.

### Analysis of Cell Cycle and Apoptosis

Cell seeding was done on six-well plate at 1 × 10^6^ cells/well and grown to 70% confluence before treatment with TR35 at 0, 1, 2, and 4 mg/ml for 24 and 48 h. Cell cycle analysis was done using propidium iodide (PI) following manufacturer instructions (cell cycle detection kit, BD, 550825). Apoptosis analysis was done using a Annexin V-FITC Apoptosis Detection Kit I (BD Biosciences, 556547) and Annexin V-APC/PI Apoptosis Kit (Sungene Biotech, AO2001-11A-H) using manufacturer protocol and analyzed by flow cytometry on FACSCalibur (BD Biosciences). The flow cytometry data were analyzed using FlowJo X.

### RNA-seq Analysis

Seeding of A549 cells was done at a density of 5 × 10^6^ cells/flask in two 75 cm^2^ flasks. Treatment with TR35 (4 mg/ml) for 48 h was done in one flask while the other was used as the untreated (control). Then, a High Pure RNA Isolation Kit (Roche, 11828665001) was used to extract Total RNA. cDNA was synthesized using RNA-Seq Sample Prep Kit (Illumina) as per the manufacturers’ instructions. Quality control analysis of the sample library was done on an Agilent 2100 Bioanalyzer and ABI StepOnePlus RT-PCR. The cDNA libraries were sequenced at the Beijing Genomics Institute (BGI, Shenzhen, China) using a HiSeq 2000 platform (Illumina).

### Protein Extraction and Western Blot

The procedures of Western blot analysis were as described previously (Tian et al., 2020). Briefly, cells were lysed using RIPA lysis buffer (Santa, sc-24948, Germany). The BCA Protein Assay Kit (Thermo Fisher Scientific, 23227) was used to determine protein concentrations, according to the manufacturer’s instructions. Protein samples were resolved by SDS-PAGE and detected by immunoblotting with antibodies against c-Myc (CST, 13987), Bcl-XL (CST, 2764), p-STAT3 (CST, 9145), STAT3 (CST, 9139S), p-JNK (CST, 4668), JNK (CST, 9252T), p-p38 (CST, 4551), and p38 (CST, 8690T). Tubulin was used as the loading control and band intensities evaluated on Image J.

### Animals and Treatment

A total of twenty 4-week old BALB/c nude mice (18–22 g) were obtained from the Beijing Vital River Laboratory Animal Technology Co., Ltd. (Beijing, China). The mice were kept in specific pathogen-free (SPF) conditions, at 23 ± 2°C, 12 h light/dark cycles at the animal facility, School of Life Sciences, Nankai University. The mice were randomly split into four groups (five mice each). Two treated groups and two control groups were given TR35 (4 mg/ml, in Milli-Q H_2_O) and an equal volume of H_2_O, respectively, by oral gavage, twice a day. After 1 week, one treated group and one control group were subcutaneously injected with 2 × 10^6^ A549 cells on the back side by axillary. The other treated and control groups were subcutaneously injected with 2 × 10^6^ H1299 cells on the back side by axillary. The body weights of mice were determined after every 3 days. Thirty days after the appearance of subcutaneous scleroma, the nude mice were sacrificed, after which tumors were harvested and weighed. The tumors were divided into two pieces for western blot and Immunohistochemical (IHC) staining analysis.

### Immunohistochemical Staining

The paraformaldehyde fixed tumor tissue were subjected to IHC staining according to standard procedures recommended by CST. Primary antibodies against p-JNK (1:200, CST, 4668), p-p38 (1:150, CST, 4551), p-STAT3 (1:200, CST 9145) diluted in 5% goat serum in 0.1% TBST were incubated with different tissues at 4°C overnight. Second and third antibody labeling was done using the rabbit anti-histochemical kit (Zhongshan Jinqiao, China), according to the manufacturer’s instructions. The IHC positive rate was determined using the H-Score method by two independent pathologists at our center.

### Statistical Analysis

Statistical testing was divided into three independent experiments, each in triplicate and data were presented as mean ± SEM. The differences between the two groups were analyzed with Student’s *t* test. The significant differences among the three groups or more than three groups were analyzed by single factor analysis of variance (ANOVA) and then compared many times by LSD test (SPSS ver. 17). *P* ≤ 0.05 was set as the threshold for statistical significance. ^∗^*p* < 0.05; ^∗∗^*p* < 0.005; ^∗∗∗^*p* < 0.001.

## Results

### TR35 Inhibits Lung Cancer Cells Proliferation

**FIGURE 1:**
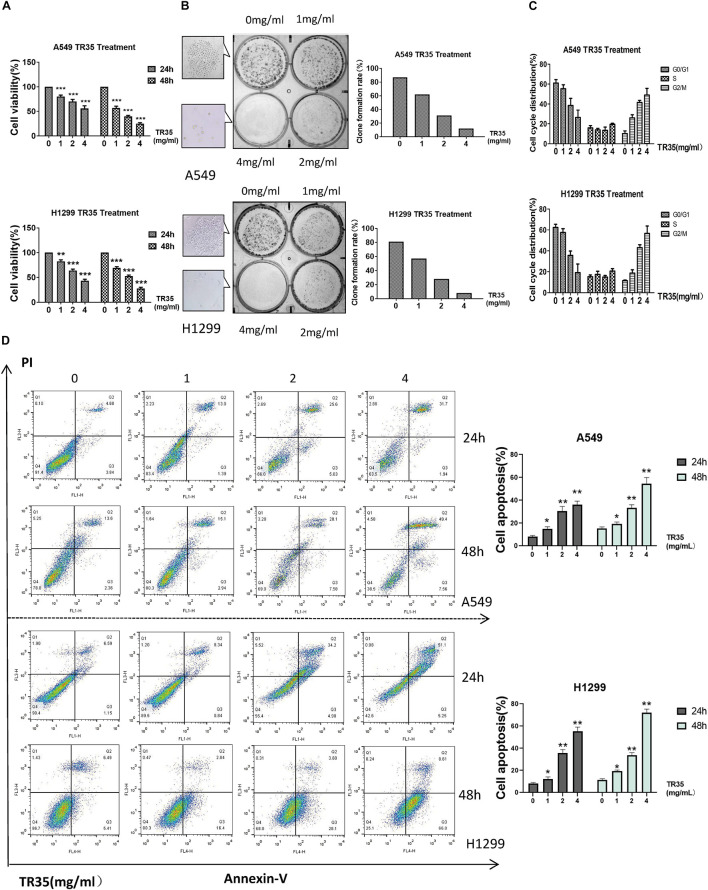
TR35 induced inhibition of A549 and H1299 cell growth. **(A)** CCK-8 assay evaluation of the inhibition of proliferation of A549 and H1299 cells incubated with TR35 at different concentrations and different times. **(B)** Colony-formation of A549 and H1299 cells inhibited by TR35. Cells were exposed to different concentrations of TR35 for 11 days in the colony formation assay. **(C)** TR35-induced G2/M cell cycle arrest. G2/M cell cycle arrest increased in the TR35 treated A549 and H1299 cells. **(D)** Flow cytometry of Annexin V and PI double staining of A549 and H1299 cells treated with TR35 at different concentrations for 24 and 48 h. Columns show the percentages of apoptotic cancer cells incubated with TR35. The data are representative of three independent experiments. Data are expressed as the mean ± SEM of three determinations (*p < 0.05, **p < 0.01, ***p < 0.001 vs. CON).

To evaluate the effects of TR35 on lung cancer cells, A549 and H1299 cells were administered with TR35 at 1, 2, and 4 mg/ml for 24 and 48 h followed by clonogenic and CCK-8 assays to assess growth and proliferation. This analysis revealed that TR35 suppressed lung cancer cells growth and proliferation in a dose-dependent manner, and these effects were most pronounced using TR35 at 4 mg/ml for 48 h (Figure 1A; inhibition rate = 76.4% in A549 and 72.5% in H1299). Examination TR35 long-term effects using clonogenic assays showed that treatment with TR35 for 12 days suppressed the colony-forming capacity of lung cancer cells (Figure 1B).

### TR35 Induced G2/M Cell Cycle Arrest and Apoptosis

Since treatment with TR35 reduced lung cancer cells viability, we assessed its effects on the cell cycle. Flow cytometry analysis showed that treatment with TR35 enhanced cell numbers in the G2/M phase and inhibited cell numbers in the G0/G1 phase (Figure 1C). Suggesting that treatment with TR35 may suppress cell growth by promoting cell cycle arrest. Additionally, treating lung cancer cells with various TR35 concentrations for 48 h significantly increased the number of cells in early or late apoptosis, while reducing the number of viable cells (Figure 1D). Together, these data show that TR35 suppresses lung cancer cell growth by inducing cycle arrest and apoptosis.

### RNA-seq

**FIGURE 2:**
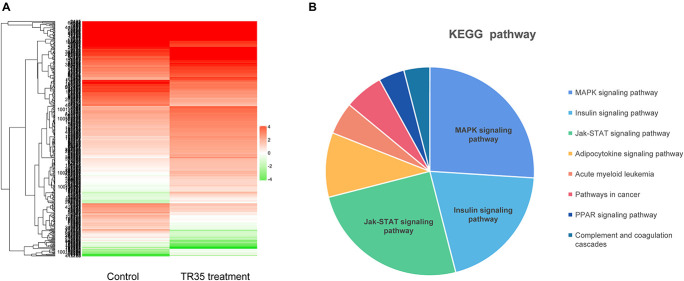
The Heatmap and KEGG pathway enrichment analysis of DEGs in A549 cells after TR35 treatment. **(A)** Heatmap of expression profile for the 433 DEGs. **(B)** KEGG pathway enrichment analysis of DEGs.

Next, RNA-seq was used to better evaluate the mechanisms driving TR35 effects on lung cancer cell growth and proliferation. To this end, a cDNA library from A549 cells treated with 4 mg/ml TR35 for 48 h and a mock-treated control library were sequenced. A total of 433 differential expression genes (DEGs) were identified (TR35-v-control), including 180 downregulated genes and 253 upregulated genes (twofold as the cut off value). The heatmap used for hierarchical clustering analysis (HCA) exhibited distinct gene expression between control and treated group (Figure 2A). KEGG pathway analysis indicated that the DEGs were enriched for eight pathways, especially MAPK and Jak-STAT signaling pathway, which were significantly different in TR35-treated cells vs. controls as determined by hypergeometric distribution (*p* ≤ 0.05, Figure 2B).

### TR35 Activates JNK and Suppresses STAT3 Signaling in Lung Cancer Cells

**FIGURE 3:**
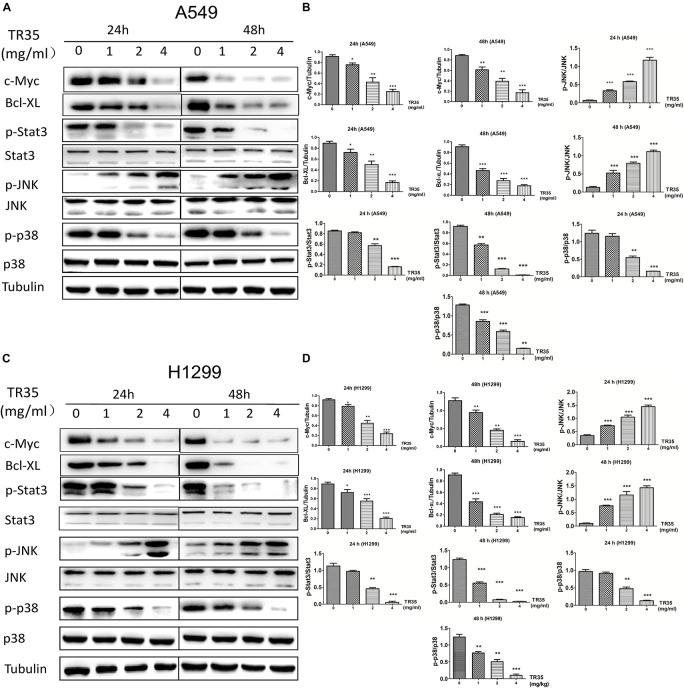
Activation of JNK and reduction of p38 and STAT3 activities by TR35 treatment in H1299 and A549 cells. **(A,C)** A549 and H1299 cells were treated with TR35 (0–4 mg/mL) for 24 and 48 h. STAT3, p38, and JNK steady state and phosphorylated levels were analyzed by western blotting. Tubulin was used as loading controls. **(B,D)** Quantification of the band density was analyzed by ImageJ software. Data are presented as the mean ± SEM, *n* = 3 (*p < 0.05, **p < 0.01, ***p < 0.001 vs. CON).

Mitogen-activated protein kinase and Jak-STAT signaling are crucial mediators of extracellular stimuli to the nucleus, modulating gene expression and thus, cell proliferation and apoptosis. RNA-seq and KEGG pathway suggested that TR35 affects lung cancer cell proliferation and apoptosis through these pathways. To test this possibility, we used western blotting to evaluate the levels of total and phosphorylated p38, JNK, and STAT3 proteins in A549 and H1299 cells treated with TR35 at 1, 2, and 4 mg/ml for 24 and 48 h. This analysis showed that TR35 dose-dependently enhanced p-JNK level while reducing p-p38 and p-STAT3 levels without significantly affecting total JNK, p38, and STAT3 protein levels. At the same time, the expression level of c-Myc and Bcl-XL, which are downstream molecules of STAT3, was also downregulated (Figures 3A–D). These results indicated that TR35 affects lung cancer cell growth, proliferation and apoptosis via MAPK and Jak-STAT signaling.

### TR35 Suppresses Non-small Cell Lung Cancer Xenograft Tumor Growth *in vivo*

**FIGURE 4:**
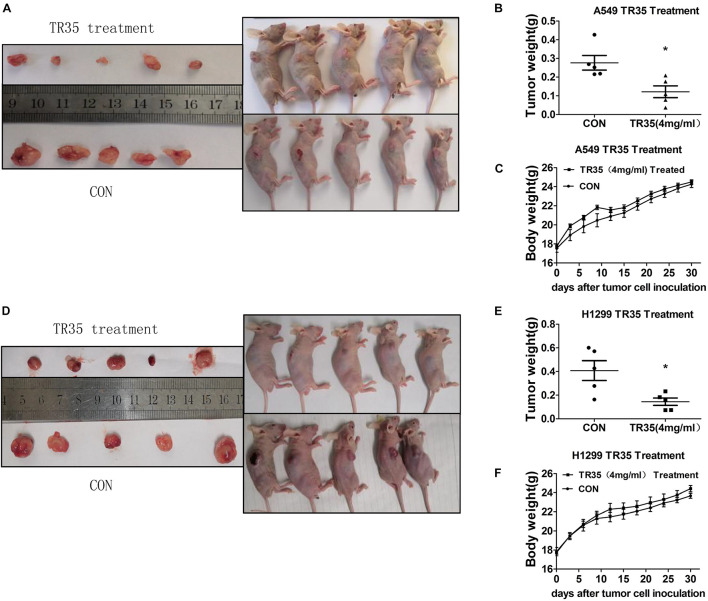
The inhibitory effect of TR35 intragastric feeding on the growth of lung cancer xenograft in nude mice. Equal amounts of A549 and H1299 cells were inoculated under the skin of the back of nude mice. **(A,D)** After TR35 treatment, the animal images of unresectable tumors and tumor images of resected tumors were obtained in animals injected with different cell lines. **(B,E)** The comparison of tumor weights of two groups in animals injected with different cell lines. **(C,F)** Animal weight was recorded once every 3 days in animals injected with different cell lines. Data are presented as the mean ± SEM, *n* = 5 (*p < 0.05 vs. CON).

To evaluate the *in vivo* effects of TR35 on NSCLC cell growth and proliferation, a xenograft mouse model was established through the subcutaneous injection of A549 and H1299 cells into SPF nude mice. Two treated groups and two control groups were treated with TR35 or a corresponding volume of H_2_O (mock), respectively. Subcutaneous tumor formation was observed by naked eye in all nude mice 7–10 days after injection. Thirty days later, tumors were harvested and their final weights taken to assess TR35 anti-tumor effects *in vivo*. This analysis found that the size and weight of tumors from TR35-treated mice were significantly lower than control tumors. However, mouse weight did not differ significantly between the groups (Figures 4A–F).

**FIGURE 5:**
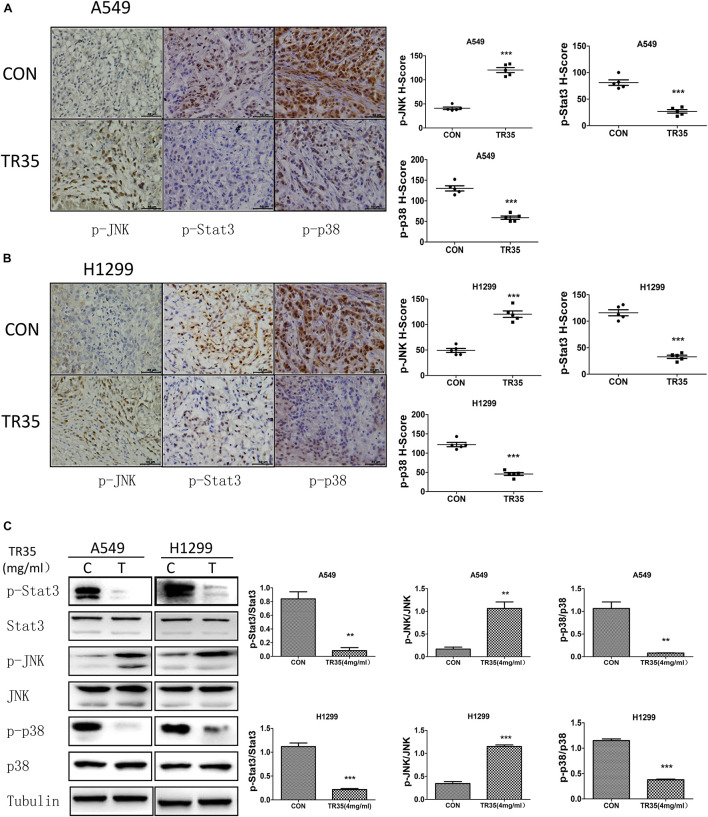
Western blotting and Immunohistochemistry were used to evaluate the effect of TR35 on the expression of p-JNK, p-p38, p-STAT3 protein in dissected tumor tissue. **(A,B)** The expressions of p-JNK, p-p38, and p-STAT3 in dissected tumor samples formed by different cell lines were measured by IHC under a light microscope. Scale bar 50 μm. **(C)** STAT3, p38, and JNK steady state and phosphorylated levels were analyzed in dissected tumor samples formed by different cell lines were measured by western blotting (**p < 0.01, ***p < 0.001 vs. CON).

Immunohistochemical analysis revealed that relative to controls, TR35-treated tumors had significantly higher p-JNK levels and significantly lower levels of p-p38 and p-STAT3 (Figures 5A,B). Similar results were obtained by western blotting (Figure 5C).

## Discussion

**FIGURE 6:**
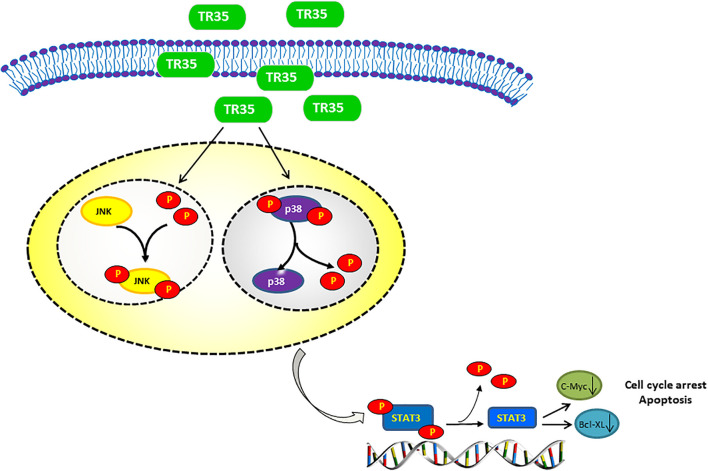
Proposed model of the mechanism underlying the TR35 inhibit NSCLC cell growth and proliferation via MAPK and Jak-STAT signaling pathway. Mechanistically, In TR35 treated NSCLC cells, JNK activation in the JNK-STAT3 signaling axis suppresses STAT3 phosphorylation accompanying the p-p38 downregulation, and thereby suppressing the growth and proliferation of NSCLC cells.

Some dietary constituents are known to be chemoprevention and multiple studies have reported dietary adjuvant therapy for cancer, including camel milk. However, few studies have examined molecular mechanisms underlying the anti-tumor effect of camel milk, especially in lung cancer. Here, we show that TR35, an active fraction obtained from Xinjiang Bactrian camel milk, effectively suppresses lung cancer cell growth by inducing apoptosis and G2/M cycle arrest. Mechanistically, we find that TR35 exerts anti-tumor effects by activating p-JNK/MAPK signaling and suppressing STAT3 signaling (including its downstream molecules such as c-Myc and Bcl-XL). However, p-p38 expression was reduced. These observations were suggested by RNA-seq data and confirmed using *in vivo* and *in vitro* assays (Figure 6).

Natural compounds are reported to induce apoptosis via MAPK signaling (Park et al., 2017; Chen and Chen, 2018). Apoptosis is influenced by a variety of intracellular proteins and complex signaling pathways. Mounting evidence implicates STAT3 and MAPK signaling in cancer cell proliferation and apoptosis (Zou et al., 2016; Meng et al., 2018). MAPK signaling pathways fall into 3 main classes: ERK, JNK and p38 (Haagenson and Wu, 2010). ERK regulates cancer cell differentiation, proliferation and apoptosis (Zhai et al., 2016). Toxic and environmental stresses activate JNK, which modulates inflammation by controlling cell differentiation, proliferation, survival, as well as migration (Park et al., 2016). Cell-stress induced signaling activates P38 in response to oxidative stress and toxic chemicals (Tsai et al., 2017). STAT3 activation promotes cell proliferation, angiogenesis, multidrug resistance, and suppresses apoptosis (Miao and Zhang, 2015). Our findings show that TR35 activates JNK signaling, while inhibiting p38 and STAT3 signaling. JNK signaling is associated with the development of numerous disorders, including cancers (Johnson and Nakamura, 2007; Weston and Davis, 2007; Wang et al., 2016). STAT3 is constitutively activated in many cancers and may be oncogenic (Kim et al., 2007). Various studies suggest STAT3 expression is elevated in tumors relative to normal tissues and its long-term activation correlates with development of various cancers (Inoue et al., 2007). Elevated STAT3 function is reported to prevent tumor cell apoptosis, while its inhibition suppresses proliferation and induces apoptosis in cancer cells (Thorburn et al., 2008; Tournier, 2013; Tan et al., 2017). This may be due to many proteins downstream of STAT3, which are crucial for tumor cell proliferation and survival were also downregulated in this process, such as c-Myc and Bcl-XL (Yu et al., 2007). JNK has been suggested as an upstream STAT3 kinase (Sun et al., 2014), and its activation in the JNK-STAT3 signaling axis suppresses STAT3 phosphorylation. JNK activation and STAT3 inhibition are reported to induce M1 macrophage polarization in lung cancer, which may have anti-tumor effects (Cui et al., 2020). These reports are consistent with our data from NSCLC cells and tumor tissues (Figures 3, 5).

Numerous studies have shown that JNK/MAPK and P38/MAPK signaling inactivate STAT3, inducing apoptosis. In this process, p-JNK and p-p38 levels are elevated. However, we observed that TR35 suppressed p-p38 levels in NSCLC cells and tumor tissues. Previous studies have reported the persistent phosphorylation of p38 and STAT3 in NSCLC. The constitutive activation of p38 and STAT3 is related to increase in cell proliferation and metastasis in NSCLC (Greenberg et al., 2002; Dutta et al., 2014; Harada et al., 2014). Greenberg et al. (2002) analyzed tissues from 20 NSCLC cases and found that the activity level of p38 was twice higher than in adjacent tissues, suggesting that p38 modulates malignant growth and transformation of cells. Additionally, Zhou et al. confirmed that reducing p-p38 levels and increasing p-JNK level inhibits cell proliferation (Zhou et al., 2020). These reports are consistent with our findings, indicating that the anti-tumor effects of p-p38 downregulation may occur via other factors, apart from p38-STAT3 signaling axis. However, the specific mechanism is unclear and further research is needed.

Cell cycle homeostasis is important in the maintenance of intracellular stability. However, cell cycle is arrested through various mechanisms, including inhibition of cyclins and expression of CDKs, when cells are damaged (Sarita Rajender et al., 2010; Bonelli et al., 2014; Cheng et al., 2017). Here, flow cytometry revealed that TR35 elevated cell numbers in the G2/M phase and suppressed cell numbers in the G0/G1 phase. Previous studies have showed that the inhibition of STAT3 signaling pathways will lead to tumor-associated G2/M phase arrest. This is because STAT3 can mediate the activity of cyclin B1/CDK protein complex (Shao et al., 2017; Yang L. et al., 2019; Nyiramana et al., 2020).

In summary, we find that TR35 inhibits NSCLC cells growth and proliferation, induces G2/M cell cycle arrest and apoptosis. This suppression process in NSCLC is via MAPK and Jak-STAT signaling. Our data highlight TR35 as a promising candidate for lung cancer therapy.

## Data Availability Statement

The datasets presented in this study can be found in online repositories. The names of the repository/repositories and accession number(s) can be found below: European Nucleotide Archive, ERS7253991 and ERS7253992.

## Ethics Statement

The animal study was reviewed and approved by the Institutional Animal Care and Use Committee of Nankai University.

## Author Contributions

ZS: writing – original draft and investigation. YG: investigation and writing – review and editing. LF: software and writing – review and editing. WT: validation and writing – review and editing. ZD, CL, JLiu, YX, YW, JYan, QW, JLi, and LY: writing – review and editing. ZZ: conceptualization and writing – review and editing. JYang: resources and writing – review and editing. ZQ: writing – original draft and funding acquisition. All authors contributed to the article and approved the submitted version.

## Conflict of Interest

The authors declare that the research was conducted in the absence of any commercial or financial relationships that could be construed as a potential conflict of interest.

## Publisher’s Note

All claims expressed in this article are solely those of the authors and do not necessarily represent those of their affiliated organizations, or those of the publisher, the editors and the reviewers. Any product that may be evaluated in this article, or claim that may be made by its manufacturer, is not guaranteed or endorsed by the publisher.
